# Pharmacokinetic Drug Interaction between Tofacitinib and Voriconazole in Rats

**DOI:** 10.3390/pharmaceutics13050740

**Published:** 2021-05-18

**Authors:** Ji-Sang Lee, Hyo-Sung Kim, Yong-Seob Jung, Hyeon-Gyeom Choi, So-Hee Kim

**Affiliations:** College of Pharmacy and Research Institute of Pharmaceutical Science and Technology, Ajou University, Suwon 16499, Korea; jis117@ajou.ac.kr (J.-S.L.); rlagytjd12@ajou.ac.kr (H.-S.K.); yongseob5730@ajou.ac.kr (Y.-S.J.); ggb1212@ajou.ac.kr (H.-G.C.)

**Keywords:** tofacitinib, voriconazole, pharmacokinetics, non-competitive inhibition, CYP3A, CYP2C, drug interaction

## Abstract

Fungal infections are prevalent in patients with immune diseases. Voriconazole, a triazole antifungal drug, inhibits the cytochromes CYP3A4 and CYP2C, and tofacitinib, a Janus kinase inhibitor for the treatment of rheumatoid arthritis, is metabolized by CYP3A4 and CYP2C19 in humans. Here, we investigated their interaction during simultaneous administration of both drugs to rats, either intravenously or orally. The area under the plasma concentration–time curve from time zero to time infinity (AUC) of tofacitinib was significantly greater, by 166% and 171%, respectively, and the time-averaged non-renal clearance (CL_NR_) of tofacitinib was significantly slower (59.5%) than that for tofacitinib alone. An in vitro metabolism study showed non-competitive inhibition of tofacitinib metabolism in the liver and intestine by voriconazole. The concentration/apparent inhibition constant (*K*_i_) ratios of voriconazole were greater than two, indicating that the inhibition of tofacitinib metabolism could be due to the inhibition of the CYP3A1/2 and CYP2C11 enzymes by voriconazole. The pharmacokinetics of voriconazole were not affected by the co-administration of tofacitinib. In conclusion, the significantly greater AUC and slower CL_NR_ of tofacitinib after intravenous and oral administration of both drugs were attributable to the non-competitive inhibition of tofacitinib metabolism via CYP3A1/2 and CYP2C11 by voriconazole in rats.

## 1. Introduction

**Figure 1:**
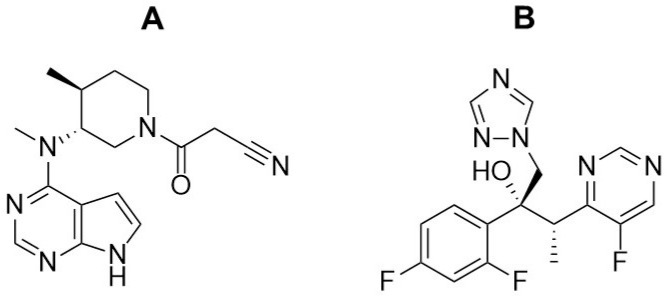
Structures of tofacitinib (**A**) and voriconazole (**B**).

Tofacitinib (Figure 1A) was developed to treat rheumatoid arthritis by inhibiting the Janus kinases (JAKs) 1 and 3 [1], which inhibits the production of inflammatory mediators and interferes with the JAK-signal transducers and activators of transcription (STAT) pathway [2,3]. Tofacitinib is preferentially prescribed to patients suffering from moderate to severe rheumatoid arthritis who are intolerant of or show a poor response to methotrexate [4,5]. Recently, tofacitinib was approved for the treatment of ulcerative colitis, making it the first oral JAK inhibitor available for ongoing treatment [6].

After the oral administration of tofacitinib to healthy subjects, pharmacokinetic analysis confirmed that its volume of distribution was 1.24 L/kg, its elimination half-life was 3.2 h, and approximately 40% of the dose showed plasma protein binding [7,8,9]. Regarding excretion, 30% of the dose was excreted in urine through the kidneys unchanged, while 70% was metabolized and excreted in the urine as metabolites [7,8,9]; the 70% metabolized tofacitinib fraction undergoes oxidation and *N*-demethylation, and comprises about 50% processed by CYP3A4 and 20% by CYP2C19. It is then further metabolized to form glucuronide conjugates [7]. The extent of the absolute oral bioavailability (*F*) value of tofacitinib is approximately 74% [9].

Unfortunately, the immunosuppression caused by long-term treatment with tofacitinib is associated with a high probability of opportunistic infection [10]. Long-term administration of tofacitinib to *Candida albicans*-infected mice has been reported to significantly reduce their survival rate [11]. In clinical practice, esophageal candidiasis, *Pneumocystis jirovecii* pneumonia, cryptococcal infection [12,13], and histoplasmosis [14] have been reported following long-term administration of tofacitinib. Once mycosis has been diagnosed in patients on long-term tofacitinib treatment, antifungal therapy should be initiated to reduce morbidity and mortality.

When tofacitinib is co-administered with antifungal agents, drug–drug interactions can be identified by observing changes in the plasma concentration of the drugs. These drug interactions are largely due to changes in drug metabolism involving cytochrome P450 (CYP); the inhibition of drug metabolism and drug interactions in humans can therefore be predicted from studies using laboratory animals.

Voriconazole (Figure 1B) is a triazole-based antifungal drug recommended as a first-line therapy for aspergillosis [15], as well as candidiasis, coccidioidomycosis, histoplasmosis, and penicilliosis [16]. Voriconazole is well absorbed from the gastrointestinal tract, with an *F* of over 90% [17]. Its half-life is estimated to be approximately 6 h and its protein binding is approximately 58% [18]. The renal excretion of unchanged voriconazole is almost negligible in humans [19]. It is extensively metabolized in the liver, mainly by CYP2C19 and, to a lesser extent, by CYP3A4 and CYP2C9, through *N*-oxidation and hydroxylation [20]. This indicates that voriconazole could cause drug–drug interactions, similar to those seen with other triazole and imidazole antifungal drugs, such as itraconazole, fluconazole, and ketoconazole.

When rheumatoid arthritis patients on long-term tofacitinib treatment also receive voriconazole, drug–drug interactions can occur, as both drugs are metabolized by the CYP3A and CYP2C subfamilies. In this study, we investigated the pharmacokinetic drug interaction between tofacitinib and voriconazole after simultaneous intravenous and oral administration of the two drugs using a rat model.

## 2. Materials and Methods

### 2.1. Chemicals

Tofacitinib citrate, voriconazole, hydrocortisone (an internal standard for tofacitinib analysis), and polyethylene glycol (PEG) 400 were purchased from Sigma-Aldrich (St. Louis, MO, USA). Heparin and 0.9% NaCl injectable solutions were purchased from JW Pharmaceutical Corporation (Seoul, Korea). Ethyl acetate for high-performance liquid chromatography (HPLC) analysis and β-cyclodextrin were purchased from J.T. Baker (Phillipsburg, NJ, USA) and Wako (Osaka, Japan), respectively. The β-nicotinamide adenine dinucleotide phosphate hydrogen (NADPH)-generating system was obtained from Corning Life Sciences (Woburn, MA, USA). Primary CYP2C11 and CYP3A1/2 antibodies were kindly donated by Detroit R&D Inc. (Detroit, MI, USA) and β-actin was purchased from Cell Signaling Technology (Beverly, MA, USA). Secondary goat, rabbit, and mouse antibodies were purchased from Bio-Rad (Hercules, CA, USA). All other chemicals and reagents were of analytical or HPLC grade and used without further purification.

### 2.2. Animals

Seven-week-old male Sprague–Dawley rats weighing approximately 230–240 g were purchased from OrientBio Korea (Seongnam, Korea) and maintained at 50 ± 5% relative humidity and 22 ± 1 °C with a 12-h (07:00–19:00)/12-h (19:00–07:00) day/night cycle. All rats were individually housed in a clean room with air purification at the Laboratory Animal Research Center of Ajou University Medical Center (Suwon, Korea) and were allowed free access to unrestricted food and water. All experimental protocols and animal manipulations were approved by the Institutional Animal Care and Use Committee (IACUC No. 2020-0018, 1 June 2020) of the Laboratory Animal Research Center of Ajou University Medical Center and were performed according to the standard operating procedures of the IACUC.

### 2.3. Intravenous and Oral Administration of Tofacitinib and Voriconazole

After each rat was anesthetized with ketamine (100 mg/kg), the jugular vein was cannulated for intravenous administration and the carotid artery for blood collection, according to previously reported methods [21,22,23]. Thereafter, during the experiment, all the rats were allowed to move freely.

For intravenous study, rats were given tofacitinib citrate (dissolved in 0.9% NaCl injectable solution containing 5% β-cyclodextrin) at a dose of 10 mg/kg (*n* = 5), voriconazole (diluted in 0.9% NaCl injectable solution containing 50% PEG 400) at a dose of 10 mg/kg (*n* = 6), or both drugs (10 mg/kg each, *n* = 6). In all cases, the treatment was administered intravenously for 1 min via the jugular vein (total injection volume of 4 mL/kg). Blood samples (approximately 0.12 mL for single drug administration and 0.22 mL for co-administration of both drugs) were taken from the carotid artery at 0 (before the start of injection), 1 (right after injection), 5, 15, 30, 60, 120, 180, 240, 360, 480, 600 and/or 720 min after the start of injection. The blood samples were centrifuged immediately and 50 μL plasma samples (one for single drug administration or two for drug co-administration) were collected and stored at −70 °C until HPLC analysis of voriconazole [24] and tofacitinib [25]. Urine samples, collected for 24 h following injection, and gastrointestinal tract (including its contents and feces) samples, collected 24 h after injection, were prepared in accordance with previously reported methods [21,22].

Tofacitinib citrate (as above) at a dose of 20 mg/kg (*n* = 6), voriconazole (as above) at a dose of 20 mg/kg (*n* = 6), or both drugs together (20 mg/kg each, *n* = 7) were orally administered using a gastric gavage (total oral volume of 6 mL/kg). Blood samples (volumes as above) were collected from the carotid artery at 0 (before oral administration) and 5, 15, 30, 60, 90, 120, 180, 240, 360, 480, 600, 720 and/or 960 min after oral administration. Plasma, urine, and gastrointestinal tract samples were obtained and processed as in the intravenous study.

### 2.4. Metabolic Inhibition of Tofacitinib by Voriconazole in Rat Hepatic Microsomes

Rat hepatic microsomes were prepared (Appendix A) and the inhibition of tofacitinib metabolism by voriconazole was measured using similar methods to those previously published [26]. Hepatic microsomes containing 0.5 mg protein and 5 μL tofacitinib (as a substrate) to final concentrations of 10, 20, 40, 80 and 200 μM; 5 μL of dimethyl sulfoxide (DMSO) containing final voriconazole (as an inhibitor) concentrations of 0, 5, 10, 20, or 40 μM; and 50 μL of 0.1 M phosphate buffer (pH 7.4) containing 1 mM NADPH were added to a test tube. The final volume was adjusted to 0.5 mL by adding 0.1 M phosphate buffer (pH 7.4). The mixture was then shaken at 50 oscillations per minute during incubation at 37 °C. Incubation times were 5, 10, 15, and 30 min. The reaction rate was within the linear range for all incubation times. After the incubation was completed, 1 mL of acetonitrile was added to terminate the reaction.

### 2.5. Estimation of Enzyme Activity in Rat Hepatic and Intestinal Microsomes

The procedures for the preparation of rat hepatic and intestinal microsomes are described in Appendix A [26,27] and the estimation of maximum velocity (*V*_max_) and the Michaelis–Menten constant (*K*_m_, concentration of substrate at a half rate of *V*_max_) as tofacitinib levels decrease, in the presence or absence of the inhibitor voriconazole, were similar to those previously published [26,27]. Each mixture was incubated with or without 20 μM voriconazole. The remaining procedures were similar to those used in the above metabolic inhibition study.

The kinetic constants (*K*_m_ and *V*_max_) for the elimination of tofacitinib with or without voriconazole were estimated using a non-linear regression method [28]. Intrinsic clearance (CL_int_) was calculated by dividing *V*_max_ by *K*_m_.

The apparent inhibition constant (*K*_i_) values of voriconazole were calculated using the following equation for non-competitive inhibition [29]:

*K*_i_ = *V*_max_’·[*I*]/(*V*_max_ − *V*_max_’) (1)

where [*I*] is the concentration of voriconazole as an inhibitor (voriconazole), and *V*_max_’ is the apparent maximum velocity of tofacitinib (substrate) with voriconazole. The apparent *K*_i_ is the concentration of voriconazole to produce a half rate of *V*_max_ for the elimination of tofacitinib. In the case of non-competitive inhibition, the slope of the inverse velocity for the elimination of a substrate against the concentration of an inhibitor decreases as the concentration of substrate increases until the slope lines meet at a point on the *x* axis; this point where they converge is −*K*_i_ [29].

### 2.6. Estimation of Protein Binding of Tofacitinib to Rat Plasma in the Absence or Presence of Voriconazole

The protein binding study of tofacitinib at a concentration of 5 μg/mL, in the absence or presence of voriconazole at a concentration of 5 μg/mL, to fresh rat plasma (*n* = 5 in each experiment) was performed using the equilibrium dialysis method [22]. Briefly, plasma was dialyzed against isotonic phosphate buffer (pH 7.4) in a 1 mL dialysis cell (Spectrum Medical Industries, Los Angeles, CA, USA). After 8 h of incubation, the concentrations of tofacitinib in both the plasma and buffer compartments were measured.

### 2.7. Measurement of Hepatic and/or Intestinal Concentrations of Voriconazole after Intravenous and Oral Administration of Both Drugs

The experimental procedures used here were similar to previously published methods [26,30]. The same doses of voriconazole and tofacitinib were administered intravenously (10 mg/kg) and orally (20 mg/kg). Blood was collected via the carotid artery at 1, 5, 15, 30, 60, and 120 min in the intravenous administration group, and 5, 15, 30, 60, 120, 180, and 240 min in the oral administration group. Each blood sample was centrifuged, and two 100 μL samples of plasma were collected. Approximately 1 g of liver (for both intravenous and oral studies) and intestine (for oral study only) was excised and blotted with tissue paper (*n* = 3 per time point of each study). Each excised tissue sample was homogenized with 4 volumes of 0.9% NaCl injectable solution, then centrifuged at 9000× *g* for 10 min. Two aliquots of 100 μL supernatant were collected. The tissue supernatant and plasma samples were stored at −70 °C until voriconazole analysis by HPLC [24].

### 2.8. Immunoblot Analysis

To confirm the inhibition of CYP2C11 and CYP3A1/2 by voriconazole in vitro and in vivo, microsomal samples were prepared in both in vitro and in vivo methods. The in vitro microsomal samples were made by treating freshly isolated rat hepatic and intestinal microsomes with voriconazole at concentrations of 5, 10, 20 and 40 μM and incubating for 30 min at 37 °C, and the in vivo microsomal samples were made by isolating the hepatic and intestinal microsomes from 3 rats 30 min after the oral administration of voriconazole at a dose of 20 mg/kg to rats. To evaluate the protein expression of CYP2C11 and CYP3A1/2, microsomal proteins (20–40 μg protein per lane) were resolved using 10% sodium dodecyl sulfate polyacrylamide gel electrophoresis (SDS-PAGE), followed by transfer to a nitrocellulose membrane at 4 °C for 1 h. To detect the protein, the membrane was incubated with a diluted primary antibody against CYP2C11 and CYP3A1/2 (1:2000) overnight at 4 °C with light shaking. The membrane was then incubated with a secondary antibody conjugated with horseradish peroxidase (diluted 1:10,000) for 1 h at room temperature. Protein expression was visualized using enhanced chemiluminescence (Bio-Rad) and captured by an Image Quant LAS 4000 Mini (GE Healthcare Life Sciences, Piscataway, NJ, USA). The expression of β-actin was used as the internal standard [21,31]. The band density was measured and compared using ImageJ 1.45 s software (NIH, Bethesda, MA, USA).

### 2.9. HPLC Analysis of Voriconazole and Tofacitinib

The concentrations of voriconazole in the biological samples were determined using a Prominence LC-20A HPLC system (Shimadzu, Kyoto, Japan) with a fluorescence detector at 254 nm and 372 nm for excitation and emission, respectively [24]. Briefly, 50 μL of biological sample was deproteinized by adding 2 volumes of methanol and 50 μL of supernatant was analyzed with a reversed-phase column (C_18_; 25 cm × 4.6 mm, 5 μm; Young Jin Biochrom, Seongnam, Korea). The mobile phase consisted of 10 mM potassium phosphate buffer (pH 6.8) with 0.01 M tetramethylethylenediamine and acetonitrile at a ratio of 55:45 (*v*/*v*), which was run at a rate of 0.5 mL/min. The temperature was maintained at 30 °C [24].

The concentrations of tofacitinib in the biological samples were also determined using a Prominence LC-20A HPLC system (Shimadzu) with a UV detector at 287 nm, and 1 μL hydrocortisone (5 mg/mL) was used as an internal standard for the HPLC analysis of tofacitinib [25]. Briefly, 50 μL of biological sample was extracted with 750 µL ethyl acetate. The organic layer was evaporated and reconstituted with 100 μL of 20% acetonitrile. The reconstituted biological sample (50 μL) was analyzed with a reversed-phase column (C_18_; 25 cm × 4.6 mm, 5 μm; Young Jin Biochrom). The mobile phase was a mixture of 10 mM ammonium acetate buffer (pH 5.0) and acetonitrile at a ratio of 69.5:30.5 (*v*/*v*) with a flow rate of 1.0 mL/min. The temperature was maintained at 40 °C [25].

### 2.10. Pharmacokinetic Analysis

To analyze the pharmacokinetic parameters of tofacitinib and voriconazole, standard methods [32] were applied using a non-compartmental analysis (WinNonlin; Pharsight Corporation, Mountain View, CA, USA). *F* is the ratio of the total area under the plasma concentration–time curve from time zero to time infinity (AUC) after oral administration to the AUC after intravenous administration. The maximum plasma concentration (*C*_max_) and time at *C*_max_ (*T*_max_) were read directly from the plasma concentration–time curves [21,22].

### 2.11. Statistical Analysis

Statistical analysis of the difference between the two unpaired means was performed by Student’s *t*-test, and a *p*-value less than 0.05 was considered to be statistically significant. All data were represented as means ± standard deviation (SD), but *T*_max_ was expressed as a median (range).

## 3. Results

### 3.1. Pharmacokinetics of Tofacitinib after Intravenous and Oral Administration of Tofacitinib without and with Voriconazole

**Figure 2:**
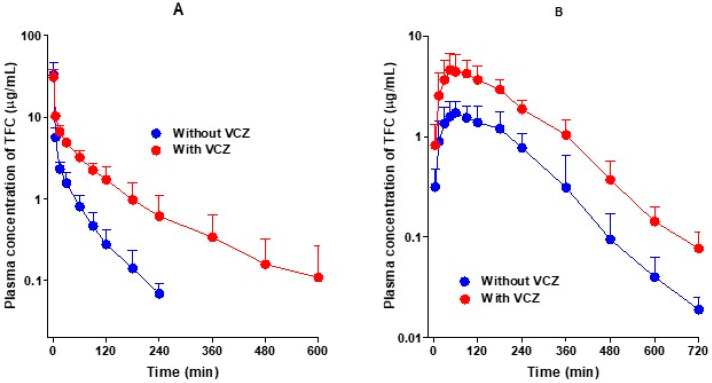
Average arterial plasma concentration–time curves of tofacitinib (TFC) after the administration of tofacitinib intravenously (**A**) without (*n* = 5) and with (*n* = 6) voriconazole (VCZ, 10 mg/kg for each drug) and orally (**B**) without (*n* = 6) and with (*n* = 7) voriconazole (20 mg/kg for each drug) to Sprague–Dawley rats. Bars represent standard deviation.

Figure 2A shows the average arterial plasma concentration–time curves of tofacitinib after the intravenous administration of tofacitinib alone and in combination with voriconazole in rats, and the corresponding pharmacokinetic parameters are summarized in Table 1. As shown in Figure 2A, the average arterial plasma concentrations of tofacitinib declined in a polyexponential manner and were significantly higher after the co-administration of voriconazole compared to when tofacitinib was administered alone. Consequently, the AUC values of tofacitinib were significantly greater (166%) in the presence of voriconazole. The changes in pharmacokinetic parameters of tofacitinib when administered in combination with voriconazole compared to the administration of tofacitinib alone are as follows. The terminal half-life and mean residence time (MRT) were significantly longer, by 141% and 220%, respectively, and the time-averaged total body (CL), renal (CL_R_), and non-renal (CL_NR_) clearances were significantly slowed, by 63.1%, 97.0%, and 59.5%, respectively. The apparent volume of distribution at steady state (*V*_ss_) and the percentage of the dose excreted unchanged in urine during the 24 h following administration (*Ae*_0–24 h_) in the two groups did not differ significantly, despite the co-administration of voriconazole. The entire gastrointestinal tract (including its contents and feces) was clear of tofacitinib 24 h after drug administration (GI_24 h_).

Figure 2B shows the average arterial plasma concentration–time curves of tofacitinib after the oral administration of tofacitinib alone and in combination with voriconazole in rats, and the corresponding pharmacokinetic parameters are summarized in Table 1. After the oral administration of tofacitinib, the absorption of tofacitinib from the gastrointestinal tract was fast, regardless of voriconazole co-administration, and tofacitinib was detected at 5 min, the first blood collection time, in both groups. The changes in the pharmacokinetic parameters of tofacitinib when orally administered in combination with voriconazole compared to the administration of tofacitinib alone are as follows. *C*_max_ and AUC were significantly greater, by 125% and 171%, respectively. In addition, *Ae*_0–24 h_ was also significantly greater, by 53.8%, but CL_R_ was significantly slower, by 42.2%, due to a greater increase in AUC than in *Ae*_0–24 h_. GI_24 h_ was also significantly greater, by 112%, but *T*_max_ did not differ significantly between the two groups.

### 3.2. Pharmacokinetics of Voriconazole after Intravenous and Oral Administration of Voriconazole without and with Tofacitinib

**Figure 3:**
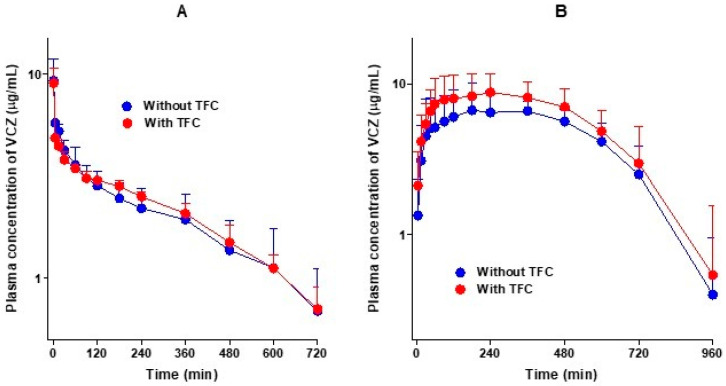
Average arterial plasma concentration–time curves of voriconazole (VCZ) after the administration of voriconazole intravenously (**A**) without (*n* = 6) and with (*n* = 5) tofacitinib (TFC, 10 mg/kg for each drug) and orally (**B**) without (*n* = 6) and with (*n* = 7) tofacitinib (20 mg/kg for each drug) to Sprague–Dawley rats. Bars represent standard deviations.

Figure 3A shows the average arterial plasma concentration–time curves of voriconazole after the intravenous administration of voriconazole alone and in combination with tofacitinib in rats, and the relevant pharmacokinetic parameters are summarized in Table 2. As shown in Figure 3A, the average arterial plasma concentrations declined polyexponentially and were comparable between the two groups, regardless of tofacitinib co-administration. The AUC values of voriconazole were therefore comparable, and not significantly different. Changes in the other pharmacokinetic parameters of voriconazole in the presence of tofacitinib compared with voriconazole alone were as follows: the terminal half-lives of voriconazole were 314 and 296 min without and with tofacitinib, respectively. The elimination of voriconazole was also comparable between the presence and absence of tofacitinib; no differences in the CL, CL_R_, and CL_NR_ of voriconazole were observed between the groups. The *Ae*_0–24 h_ values were 3.70% and 4.54% in the presence and absence of tofacitinib, respectively, indicating that most of the intravenously administered voriconazole was metabolized. However, the *V*_ss_ of voriconazole in the presence of tofacitinib was significantly greater (127%) than that in the absence of tofacitinib. Overall, tofacitinib did not have a significant effect on the pharmacokinetics of voriconazole after the intravenous administration of voriconazole in the presence or absence of tofacitinib.

Figure 3B shows the average arterial plasma concentration–time curves of voriconazole after the oral administration of voriconazole alone and in combination with tofacitinib in rats, and the relevant pharmacokinetic parameters are summarized in Table 2. After the oral administration of voriconazole, the absorption of voriconazole from the gastrointestinal tract was rapid, irrespective of whether it was accompanied by tofacitinib, and voriconazole was detected at 5 min, the first blood collection time, in both groups. Changes in the pharmacokinetic parameters of voriconazole in combination of tofacitinib compared to voriconazole alone are as follows: the *Ae*_0–24 h_ of voriconazole was significantly lower, by 71.0%, and the CL_R_ of voriconazole was significantly slower, by 87.8%. The GI_24 h_ was significantly greater in the co-administration group compared to the voriconazole-only group (1.03% vs. 0.0413% of the oral dose). However, the AUC, *C*_max_, and *T*_max_ did not differ significantly between the groups, indicating that there was no significant effect on the pharmacokinetics of voriconazole when co-administered orally with tofacitinib.

### 3.3. Non-Competitive Inhibition of Tofacitinib Metabolism by Voriconazole in Rat Hepatic Microsomes

**Figure 4:**
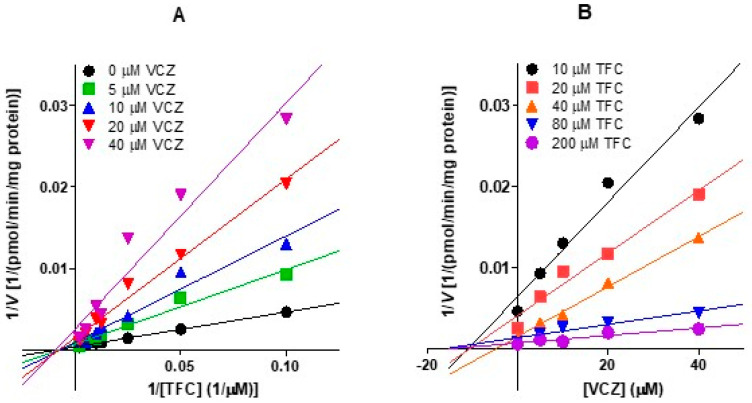
Lineweaver–Burk plots (**A**) and Dixon plots (**B**) showing the inhibition of elimination of tofacitinib by voriconazole in rat hepatic microsomes. TFC; tofacitinib, VCZ; voriconazole.

To confirm the kinetics of inhibition of tofacitinib metabolism by voriconazole, the rates for the elimination of tofacitinib from rat hepatic microsomes were measured in the absence or presence of voriconazole. The Lineweaver–Burk plot [33] illustrating these data is shown in Figure 4A. A linear relationship was observed between the reciprocal of the substrate (tofacitinib) concentration and the reciprocal of the elimination rate of tofacitinib. In addition, as the concentration of inhibitor (voriconazole) increased, the slope between the inverse of the substrate concentration and the inverse of the elimination rate of substrate increased, and the *x*-intercepts met at a point, suggesting that the inhibition of tofacitinib metabolism by voriconazole occurs in a non-competitive manner. The Dixon plot [34] for various tofacitinib concentrations also showed the linear relationship between the inhibitor concentration and the reciprocal of the velocity, and the result was met at one point on the *x*-axis (Figure 4B). This also demonstrates that the metabolism of tofacitinib is non-competitively inhibited by voriconazole in the liver.

### 3.4. V_max_, K_m_ and CL_int_ for the Elimination of Tofacitinib in the Absence or Presence of Voriconazole and Apparent K_i_ of Voriconazole for the Inhibition of Tofacitinib Metabolism in Rat Hepatic and Intestinal Microsomes

Table 3 summarizes the *V*_max_, *K*_m_, and CL_int_ values for the elimination of tofacitinib in the absence or presence of voriconazole in rat hepatic and intestinal microsomes. The *K*_m_ values in these two scenarios were similar, suggesting that the *K*_m_ of tofacitinib was not affected by voriconazole in rat hepatic and intestinal microsomes. In other words, the enzyme affinity for tofacitinib was not affected by voriconazole in this environment. However, the *V*_max_ demonstrated significantly slower removal of tofacitinib in the presence of voriconazole, by 59.1% and 44.2% in rat hepatic and intestinal microsomes, respectively, than in its absence. This suggests that the metabolism of tofacitinib was significantly inhibited by voriconazole. Thus, the CL_int_ of tofacitinib was also significantly slower in the presence of voriconazole, by 65.5% and 21.1% in rat hepatic and intestinal microsomes, respectively. The apparent *K*_i_ values of voriconazole for the inhibition of tofacitinib metabolism were estimated to be 6.52 (5.59–7.19) and 26.2 (20.2–35.3) μM in rat hepatic and intestinal microsomes, respectively. These results suggest that the inhibition of tofacitinib metabolism by voriconazole in rat hepatic and intestinal microsomes occurred in a non-competitive manner.

### 3.5. Rat Plasma Protein Binding of Tofacitinib without and with Voriconazole to Fresh Rat Plasma

The protein binding (bound fraction) values of tofacitinib (5 μg/mL) in the presence and absence of voriconazole (5 μg/mL) to fresh rat plasma were 36.1 ± 3.32% and 37.9 ± 1.57%, respectively; the values were comparable in the presence and absence of voriconazole.

### 3.6. Hepatic and Intestinal Concentrations of Voriconazole after Intravenous and Oral Co-Administration of Both Tofacitinib and Voriconazole

After both tofacitinib and voriconazole were co-administered to rats, either intravenously or orally, the concentrations of voriconazole ([*I*]) in the liver and intestine were measured and are shown in Table 4. After intravenous administration, the hepatic [*I*] up to 120 min post-treatment was higher than its hepatic *K_i_* (6.52 μM). The [*I*]/*K_i_* ratios in the liver were 3.68, 2.22, 2.56, 2.10, 1.96, and 2.30 at 1, 5, 15, 30, 60, and 120 min post-treatment, respectively (Table 4). The [*I*]/*K_i_* ratios of voriconazole in the liver were greater than or equal to two until 120 min after the intravenous administration of both drugs (Table 4). After the oral administration of both drugs, the [*I*] values of voriconazole in the liver up to 240 min post-treatment were higher than the hepatic *K_i_* of voriconazole (6.52 μM), but the values in the intestine were less than the intestinal *K_i_* of voriconazole (26.2 μM). The [*I*]/*K_i_* ratios in the liver were greater than two and the ratios in the intestine were less than two (0.158–0.447) up to 240 min (Table 4). These results suggest that the inhibition of tofacitinib metabolism by voriconazole in the liver of rats is mainly caused by the non-competitive inhibition of CYP isozymes [35]. However, it is necessary to further confirm whether the decrease in tofacitinib metabolism in the intestine is due to CYP inhibition.

### 3.7. Protein Expression of CYP2C11 and CYP3A1/2

**Figure 5:**
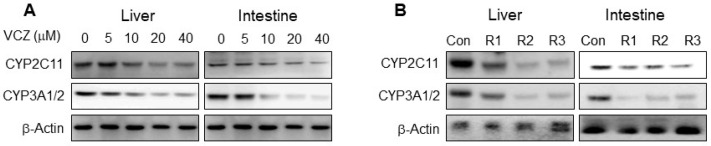
Protein expression of CYP2C11 and CYP3A1/2 in hepatic and intestinal microsomes in vitro (**A**) and in vivo (**B**). For in vitro samples, freshly isolated rat hepatic and intestinal microsomes were treated with voriconazole (VCZ) at concentrations of 5, 10, 20 and 40 μM and incubated for 30 min at 37 °C. For in vivo samples, the hepatic and intestinal microsomes were obtained from 3 rats 30 min after the oral administration of voriconazole at dose of 20 mg/kg to rats. β-Actin was used as a loading control.

Rat hepatic and intestinal microsomes were treated in vitro for 30 min without and with voriconazole at 5, 10, 20 and 40 μM, and CYP2C11 and CYP3A1/2 expression levels were shown to be significantly lower in the presence of voriconazole (Figure 5A). Similarly, the expression levels of these proteins in hepatic and intestinal microsomes obtained 30 min after oral administration of voriconazole (20 mg/kg) to rats were also significantly lower (Figure 5B). These results suggest that voriconazole affects the expression of CYP2C11 and CYP3A1/2, resulting in changes in the metabolism of tofacitinib.

## 4. Discussion

The intravenous and oral doses of voriconazole and tofacitinib administered to rats in this study (10 and 20 mg/kg, respectively) were determined based on the results of previous studies; intravenous and oral doses with a linear relationship between doses and AUC values of tofacitinib [22] and voriconazole [36] were selected.

The changes in the CL_NR_ of tofacitinib have been reported to be due to changes in tofacitinib metabolism in rats [22]. Therefore, the changes in CL_NR_ of tofacitinib (Table 1) after the intravenous co-administration of voriconazole and tofacitinib also indicate such metabolic changes. After the intravenous administration of both drugs, the CL_NR_ of tofacitinib was significantly slower than after the intravenous administration of tofacitinib alone (Table 1). Tofacitinib is a drug with an intermediate hepatic extraction ratio (42.0%) in rats [22]. Therefore, the hepatic metabolism of tofacitinib is determined by the hepatic CL_int_ for the metabolism of tofacitinib and the hepatic blood flow rate [37]. The significantly slower CL_NR_ of tofacitinib when co-administered with voriconazole (Table 1) was consistent with the significantly slower CL_int_ for the elimination of tofacitinib in the presence of voriconazole in vitro (Table 3). The slower CL_int_ of tofacitinib (Table 3) was due to the non-competitive inhibition of tofacitinib metabolism by voriconazole via CYP3A1/2 and CYP2C11 in the liver (Figure 5). The free fraction of tofacitinib in rat plasma was not affected by voriconazole, and no effect of voriconazole on the hepatic blood flow rate has been reported in rats.

The significantly slower CL_NR_ of tofacitinib after intravenous co-administration with voriconazole (Table 1) was also consistent, with the apparent *K*_i_ values of voriconazole and [*I*]/*K*_i_ ratios for the inhibition of tofacitinib metabolism in the liver up to 120 min (Table 4). Bachmann and Lewis [35] reported that for inhibitory drug–drug interactions, if the [*I*]/*K*_i_ ratio is greater than two, the drug–drug interaction is attributable to CYP inhibition, and if the ratio is less than 0.01, it is not due to CYP inhibition. The [*I*]/*K*_i_ ratio of voriconazole in the liver was found to be greater than two (Table 4), indicating that the significantly slower CL_NR_ of tofacitinib (i.e., a significant increase in AUC) was due to the inhibition of the hepatic metabolism of tofacitinib through CYP3A1/2 and CYP2C11 by voriconazole (Figure 5).

After the oral co-administration of these drugs, the AUC of tofacitinib significantly increased compared to after the oral administration of tofacitinib alone (Table 1). However, this increase in AUC does not seem to have increased the gastrointestinal absorption of tofacitinib in the presence of voriconazole. Using the equation proposed by Lee and Chiou [38], the average ‘true’ fractions of the oral dose of tofacitinib unabsorbed (‘*F*_unabs_’) were estimated in the absence or presence of voriconazole, with values of approximately 0.162% and 0.520%, respectively. Therefore, more than 99% of the oral dose of tofacitinib was absorbed, whether or not voriconazole was present.

The significantly greater AUC of tofacitinib after the oral administration of both drugs appears to be due to the non-competitive inhibition of tofacitinib metabolism by voriconazole in the liver and intestine (Table 3 and Figure 4). These results are clearly demonstrated by the decrease in CL_int_ of tofacitinib by voriconazole, the apparent *K*_i_ of voriconazole, the increase in plasma concentration of tofacitinib, and the [*I*]/*K*_i_ ratio of voriconazole in the liver and intestine. As mentioned earlier, voriconazole inhibited the hepatic metabolism of tofacitinib when both drugs were administered intravenously. Therefore, the effect of the voriconazole-induced hepatic metabolic inhibition of tofacitinib on the AUC of orally administered tofacitinib does not seem to be minor compared to the effect of the intestinal metabolic inhibition of tofacitinib by voriconazole, since the gastrointestinal first-pass effect of tofacitinib is approximately 46.1% of the oral dose, whereas the hepatic first-pass effect of tofacitinib is 21.3% of the oral dose after oral administration in rats [22]. The [*I*]/*K*_i_ ratios for the inhibition of tofacitinib metabolism in the liver up to 240 min after the oral administration of both drugs were greater than two, but the [*I*]/*K*_i_ ratios in the intestine were less than two (Table 4). This could be due to the fact that the [*I*] values were obtained from the serosa and not from the enterocytes. Since orally administered drugs are absorbed through enterocytes, a high concentration of a drug during absorption is expected in the enterocytes [39]. In addition, most intestinal CYP enzymes are located in enterocytes. In particular, CYP3A accounts for 82% of total intestinal CYP enzymes [39,40]. In this study, if the [*I*] values had been measured in enterocytes, the [*I*] values would be high and the [*I*]/*K*_i_ ratios could thus be greater than two in the intestine. Therefore, the significant increase in AUC of tofacitinib (Table 1) after the oral administration of both drugs appears to be primarily due to the non-competitive inhibition of both the intestinal and hepatic metabolism of tofacitinib (Table 3) via the inhibition of CYP3A1/2 and CYP2C11 by voriconazole in rats (Figure 5). Overall, the higher AUC of tofacitinib (171%) after the oral co-administration of both drugs was comparable to that (166%) after intravenous co-administration of both drugs. This seems to be due to the fact that the metabolic inhibition of tofacitinib by voriconazole in both the liver and intestine plays a role in the AUC increase of tofacitinb when co-administered orally.

The CL_R_ of tofacitinib was significantly slower in the presence of voriconazole after either intravenous or oral co-administration of both drugs, which is attributable to the significantly greater AUC of tofacitinib, due to the slower hepatic and intestinal metabolism of tofacitinib in both routes of administration (Table 1). Tofacitinib is mainly excreted in urine via an organic cation transporter [41]; however, the *Ae*_0–24 h_ values of tofacitinib were comparable in the absence and presence of voriconazole for both intravenous and oral routes, indicating that voriconazole does not inhibit the organic cation transporter for the excretion of tofacitinib. Therefore, the slower CL_R_ of tofacitinib when co-administered with voriconazole is attributable to the greater AUC of tofacitinib, in turn due to the metabolic inhibition of tofacitinib caused by voriconazole.

In reports on drug interactions with voriconazole, a mixed inhibition of CYP enzymes in rat hepatic microsomes was reported when it was combined with vonoprazan, a reversible potassium-competitive acid blocker, which is metabolized by CYP2B6, CYP3A4, CYP2C19, and CYP2D6 [42]. In addition, voriconazole, although not a CYP enzyme, has shown non-competitive inhibition of human carbonic anhydrase 1 in its interaction with acetazolamide, and non-competitive inhibition of α-glycosidase in its interaction with acarbose, a drug used for reducing postprandial glucose in type 2 diabetes mellitus [43]. In the case of tofacitinib, when administered orally to healthy male adults, the AUC and *C*_max_ of tofacitinib were 103% and 16% higher, respectively, when it was combined with ketoconazole, an imidazole antifungal drug, and its AUC and *C*_max_ were 79% and 27% higher, respectively, when co-administered with fluconazole, a triazole antifungal drug [44]. Therefore, dosage adjustment of tofacitinib is recommended when co-administering it with moderate to strong CYP3A4 inhibitors.

## 5. Conclusions

In summary, after the intravenous administration of tofacitinib and voriconazole to rats, the CL_NR_ of tofacitinib was significantly slower than that of tofacitinib alone (Table 1). This could be due to the non-competitive inhibition of the hepatic metabolism of tofacitinib via CYP3A1/2 and CYP2C11 by voriconazole in rats. After the oral administration of both drugs at the same time, the significant increase in AUC of tofacitinib compared to tofacitinib alone (Table 1) could be mainly due to the non-competitive inhibition of the intestinal metabolism of tofacitinib via intestinal CYP3A1/2 and CYP2C11, as well as the non-competitive inhibition of hepatic metabolism of tofacitinib via hepatic CYP3A1/2 and CYP2C11 by voriconazole. The above results will be very useful for future investigations into tofacitinib–voriconazole interactions, especially in designing clinical trials and predicting pharmacokinetic changes. In addition, if the current results obtained from rats are extrapolated to humans, the dosage regimen of tofacitinib should be modified to accommodate the decrease in metabolism of tofacitinib in chronic repeated administration, and additional clinical studies are necessary to confirm the above results.

## Figures and Tables

**Table 1:** Pharmacokinetic parameters of tofacitinib after intravenous (10 mg/kg) and oral (20 mg/kg) administration of tofacitinib without and with voriconazole (VCZ, 10 and 20 mg/kg for intravenous and oral administration, respectively) to Sprague–Dawley rats. Values are means ± standard deviation (SD).

Parameters	Intravenous		Oral	
	Without VCZ (*n* = 5)	With VCZ (*n* = 6)	Without VCZ (*n* = 6)	With VCZ (*n* = 7)
Body weight (g)	277 ± 4.55	283 ± 10.4	279 ± 6.15	282 ± 7.06
AUC (µg·min/mL)	278 ± 76.6	740 ± 154 ***	396 ± 61.6	1074 ± 271 ***
*C*_max_ (µg/mL)			1.98 ± 0.405	4.46 ± 2.00 *
*T*_max_ (min)			87.5 ± 52.3	62.5 ± 29.1
Terminal half-life (min)	43.2 ± 7.45	104 ± 30.2 **		
MRT (min)	32.8 ± 13.4	105 ± 46.7 **		
CL (mL/min/kg)	37.9 ± 8.87	14.1 ± 3.16 ***		
CL_R_ (mL/min/kg)	3.61 ± 0.829	0.144 ± 0.0678 ***	0.536 ± 0.135	0.310 ± 0.117 **
CL_NR_ (mL/min/kg)	34.3 ± 8.10	13.9 ± 3.18 ***		
*V*_ss_ (mL/kg)	1211 ± 489	1292 ± 193		
*Ae*_0–24 h_ (% of dose)	9.60 ± 1.00	10.9 ± 6.25	10.6 ± 3.49	16.3 ± 5.74 *
GI_24 h_ (% of dose)			0.411 ± 0.313	0.912 ± 0.527
*F* (%)			71.2	72.4

* Significantly different from respective control group (*p* < 0.05). ** Significantly different from respective control group (*p* < 0.01). *** Significantly different from respective control group (*p* < 0.001).

**Table 2:** Pharmacokinetic parameters of voriconazole after intravenous (10 mg/kg) and oral (20 mg/kg) administration of voriconazole without and with tofacitinib (TFC, 10 and 20 mg/kg for intravenous and oral administration, respectively) to Sprague–Dawley rats. Values are means ± standard deviation (SD).

Parameters	Intravenous		Oral	
	Without TFC (*n* = 6)	With TFC (*n* = 5)	Without TFC (*n* = 6)	With TFC (*n* = 7)
Body weight (g)	298 ± 14.9	283 ± 11.5	274 ± 4.59	283 ± 8.25
AUC (µg·min/mL)	1862 ± 600	1804 ± 436	3938 ± 416	4415 ± 760
*C*_max_ (µg/mL)			7.82 ± 1.13	8.97 ± 1.73
*T*_max_ (min)			263 ± 201	283 ± 234
Terminal half-life (min)	314 ± 96.7	296 ± 110		
MRT (min)	422 ± 132	400 ± 149		
CL (mL/min/kg)	6.01 ± 2.47	5.77 ± 1.18		
CL_R_ (mL/min/kg)	0.028 ± 0.015	0.022 ± 0.015	0.37 ± 0.24	0.096 ± 0.046 *
CL_NR_ (mL/min/kg)	5.98 ± 2.46	5.74 ± 1.17		
*V*_ss_ (mL/kg)	2275 ± 159	5169 ± 248		
*Ae*_0–24 h_ (% of dose)	4.54 ± 1.30	3.70 ± 2.43	7.48 ± 5.15	2.17 ± 1.26 *
GI_24 h_ (% of dose)			0.041 ± 0.017	1.03 ± 0.911 *
*F* (%)			106	122

* Significantly different from respective control group (*p* < 0.05).

**Table 3:** Measurement of *K*_m_, *V*_max_, CL_int_ and *K*i for the elimination of tofacitinib in the presence or absence of voriconazole (VCZ) in rat hepatic and intestinal microsomes. Values are means ± standard deviation (SD).

Parameter	Withou VCZ (*n* = 3)	With VCZ (*n* = 3)
Liver		
*K*_m_ (μM)	124 ± 13.7	147 ±43.4
*V*_max_ (pmol/min/mg protein)	2895 ± 319	1184 ± 470 ***
CL_int_ (mL/min/mg protein)	0.0233 ± 0.000689	0.00805 ± 0.00201 ***
*K*_i_ (μM)		6.52 ± 0.828
Intestine		
*K*_m_ (μM)	43.1 ± 11.7	30.1 ± 3.29
*V*_max_ (pmol/min/mg protein)	380 ± 86.1	212 ± 27.0 *
CL_int_ (mL/min/mg protein)	0.00894 ± 0.00100	0.00705 ± 0.000120 *
*K*_i_ (μM)		26.2 ± 8.05

* Significantly different from respective control group (*p* < 0.05). *** Significantly different from respective control group (*p* < 0.001).

**Table 4:** Mean ± standard deviation (SD) concentration (μM) of voriconazole in the liver and intestine after the intravenous and oral administration of voriconazole and tofacitinib to rats.

Intravenous			Oral				
Time (min)	Liver Concentration (μM)	[*I*]/*K*_i_	Time (min)	Liver Concentration (μM)	[*I*]/*K*_i_	Intestine Concentration (μM)	[*I*]/*K*_i_
1	24.0 ± 9.86	3.68	5	31.7 ± 7.91	4.86	11.7 ± 8.11	0.447
5	14.5 ± 8.31	2.22	15	22.1 ± 4.05	3.39	4.13 ± 1.65	0.158
15	16.7 ± 4.08	2.56	30	35.8 ± 5.22	5.49	10.2 ± 9.13	0.389
30	13.7 ± 2.68	2.10	60	27.6 ± 5.07	4.23	4.63 ± 0.318	0.177
60	12.8 ± 4.51	1.96	120	28.9 ± 3.01	4.43	5.08 ± 0.863	0.194
120	15.0 ± 1.01	2.30	180	32.9 ± 3.36	5.05	4.93 ± 2.21	0.188
			240	27.5 ± 4.90	4.22	4.57 ± 0.664	0.174

## Appendix A. Preparation of Rat Hepaptic and Intestinal Microsomes

The 1 g of liver was homogenized with 4 volumes of ice-cold homogenizing buffer (0.154M KCl/50 mM Tris–HCl in 1 mM EDTA, pH 7.4). The homogenate was centrifuged at 10,000× *g* for 30 min at 4 °C and the supernatant was further centrifuged at 100,000× *g* for 90 min at 4 °C. The microsomal pellet was resuspended in the homogenizing buffer.

Approximately the first two-thirds of the small intestine were excised and rinsed twice with ice-cold 10 mM potassium phosphate buffer with 1.15% KCl (pH 7.4) to remove the contents in the lumen. Then, the intestine was filled with solution A (1.5 mM KCl + 96 mM NaCl + 27 mM sodium citrate + 8 mM KH_2_PO_4_ + 5.6 mM Na_2_HPO_4_ + 40 μg/mL PMSF) and incubated at 37 °C for 15 min. After the incubation, the solution was discarded and filled with ice-cold solution B (phosphate-buffered saline + 1.5 mM EDTA + 0.5 mM dithiothreitol + 40 μg/mL PMSF). The lumen was wound around a middle finger and tapped gently against the finger. The upper villus cells were released into solution B during this process. The tapping and harvesting of cells in solution B were carried out three times for each small intestine, and the solution B with cells were pooled. The pooled solution B was centrifuged at low speed (200 rpm) for 5 min at 4 °C. The supernatant was discarded, and approximately 20 mL of ice-cold solution C (5 mM histidine + 0.25 M sucrose + 0.5 mM EDTA + 40 μg/mL PMSF) was added into each centrifuge tube; then the tube was inverted twice. Following a second centrifugation, the supernatant was discarded, and the cells were resuspended in fresh ice-cold solution C (repeated twice). Then the cells in solution C were homogenized and the homogenate was centrifuged at 10,000× *g* for 20 min at 4 °C. The supernatant was further centrifuged at 100,000× *g* for 65 min at 4 °C. The microsomal pellet was resuspended in 0.2 mM EDTA/20% glycerol/80% 0.1 M phosphate buffer (pH 7.4).
